# Induction of Mucosal IgA–Mediated Protective Immunity Against Nontypeable *Haemophilus influenzae* Infection by a Cationic Nanogel–Based P6 Nasal Vaccine

**DOI:** 10.3389/fimmu.2022.819859

**Published:** 2022-07-06

**Authors:** Rika Nakahashi-Ouchida, Hiromi Mori, Yoshikazu Yuki, Shingo Umemoto, Takashi Hirano, Yohei Uchida, Tomonori Machita, Tomoyuki Yamanoue, Shin-ichi Sawada, Masashi Suzuki, Kohtaro Fujihashi, Kazunari Akiyoshi, Yuichi Kurono, Hiroshi Kiyono

**Affiliations:** ^1^Division of Mucosal Vaccines, International Research and Development Center for Mucosal Vaccines, The Institute of Medical Science, The University of Tokyo, Tokyo, Japan; ^2^Division of Mucosal Immunology, IMSUT Distinguished Professor Unit, The Institute of Medical Science, The University of Tokyo, Tokyo, Japan; ^3^Department of Human Mucosal Vaccinology, Chiba University Hospital, Chiba, Japan; ^4^HanaVax Inc., Tokyo, Japan; ^5^Faculty of Medicine, Department of Otorhinolaryngology, Head and Neck Surgery, Oita University, Oita, Japan; ^6^CU-UCSD Center for Mucosal Immunology, Allergy and Vaccines (cMAV), Division of Gastroenterology, Department of Medicine, University of California, San Diego, San Diego, CA, United States; ^7^Department of Polymer Chemistry, Faculty of Engineering, Kyoto University, Kyoto, Japan; ^8^Division of Clinical Vaccinology, International Research and Development Center for Mucosal Vaccines, The Institute of Medical Science, The University of Tokyo, Tokyo, Japan; ^9^Department of Pediatric Dentistry, The University of Alabama at Birmingham, Birmingham, AL, United States; ^10^Department of Otolaryngology, Faculty of Medicine, Kagoshima University, Kagoshima, Japan; ^11^Future Medicine Education and Research Organization, Mucosal Immunology and Allergy Therapeutics, Institute for Global Prominent Research, Chiba University, Chiba, Japan

**Keywords:** cCHP nanogel, drug delivery system, nasal vaccine, nontypeable *Haemophilus influenzae*, mucosal IgA

## Abstract

Nontypeable *Haemophilus influenzae* (NTHi) strains form a major group of pathogenic bacteria that colonizes the nasopharynx and causes otitis media in young children. At present, there is no licensed vaccine for NTHi. Because NTHi colonizes the upper respiratory tract and forms biofilms that cause subsequent infectious events, a nasal vaccine that induces NTHi-specific secretory IgA capable of preventing biofilm formation in the respiratory tract is desirable. Here, we developed a cationic cholesteryl pullulan–based (cCHP nanogel) nasal vaccine containing the NTHi surface antigen P6 (cCHP-P6) as a universal vaccine antigen, because P6 expression is conserved among 90% of NTHi strains. Nasal immunization of mice with cCHP-P6 effectively induced P6-specific IgA in mucosal fluids, including nasal and middle ear washes. The vaccine-induced P6-specific IgA showed direct binding to the NTHi *via* the surface P6 proteins, resulting in the inhibition of NTHi biofilm formation. cCHP-P6 nasal vaccine thus protected mice from intranasal NTHi challenge by reducing NTHi colonization of nasal tissues and eventually eliminated the bacteria. In addition, the vaccine-induced IgA bound to different NTHi clinical isolates from patients with otitis media and inhibited NTHi attachment in a three-dimensional *in vitro* model of the human nasal epithelial surface. Therefore, the cCHP-P6 nanogel nasal vaccine induced effective protection in the airway mucosa, making it a strong vaccine candidate for preventing NTHi-induced infectious diseases, such as otitis media, sinusitis, and pneumonia.

## Introduction

Nontypeable *Haemophilus influenzae* (NTHi) is a human-specific pathogen that mainly colonizes the upper respiratory tract and causes noninvasive infections, including otitis media, sinusitis, and pneumonia; NTHi also is associated with exacerbation of chronic obstructive pulmonary disease (1, 2). Unfortunately, no licensed vaccine specific for NTHi infections is currently available. A licensed pneumococcal vaccine containing the protein D of NTHi, PHiD-CV (*Synflorix*, GSK), has been used in the clinical setting, but it provides very limited protection against otitis media caused by NTHi infections (3–5). In addition, the introduction of pneumococcal vaccines such as PCV13 (*Prevnar*13, Pfizer Inc.) has been suggested to have led to an increase of acute otitis media and the emergence of invasive NTHi (6). Owing to the increasing number of antibiotic-resistant NTHi strains (7), the development of NTHi vaccines has become a very important issue for public health.

NTHi colonization of the upper respiratory tract is an important first step in the pathogenesis of NTHi-mediated disease; NTHi forms biofilms that promote persistence within the host environment, and this leads to increased antimicrobial resistance (8, 9). A strategy for suppressing bacterial invasion and colonization of the mucosa of the upper respiratory tract and for inhibiting biofilm formation in the airway mucosa would, therefore, be an effective way to prevent NTHi infection.

Nasal immunization efficiently induces an antigen-specific immune response on mucosal surfaces of the upper and lower respiratory tracts, as well as in the systemic compartment (10, 11). Indeed, nasal immunization with a vaccine antigen targeting the NTHi surface antigen P6 induces both antigen-specific serum IgG and mucosal secretory immunoglobulin A (SIgA), which directly recognizes and eliminates NTHi in the nasal or bronchial mucosa, thereby preventing the initiation of infections (12, 13). However, the nasal cavity has a well-developed mucosal barrier that includes cilia, mucus secretions, and tight junctions and plays an important role in host defense through innate immunity (14). Intranasally administered vaccine antigens are easily eliminated by the mucosal barrier system, making it difficult for them to induce substantial antigen-specific immune responses. Therefore, the system used to deliver the nasal vaccine needs to overcome this obstacle. Given the nature of *Haemophilus* infections, it is logical and desirable to develop nasal vaccines that effectively activate the airway mucosal immune system, where the first line of defense against NTHi infections occurs. We therefore applied our cationic cholesteryl-group-bearing pullulan (cCHP) nanogel–based nasal delivery system to the development of a nasal vaccine against NTHi.

The cCHP nanogel is a safe and effective nasal vaccine delivery vehicle that can optimally deliver vaccine antigen and stimulate the nasal mucosal immune system (15–18). Because of its cationic property, the cCHP nanogel shows persistent attachment to the surfaces of the negatively charged nasal mucosa, leading to the prolonged release of antigen to the antigen-sampling and -presenting systems of the nasal mucosa (15). The cCHP nanogel has thus been shown to effectively induce antigen-specific immune responses in both the systemic and mucosal compartments (16–18), and we therefore believe that it is an attractive and competent vehicle for delivering nasal vaccines to prevent respiratory infectious diseases, including otitis media, pneumonia, and COVID-19. Indeed, we have demonstrated that a cCHP nanogel incorporating a pneumococcal surface protein antigen (cCHP-PspA) induces PspA-specific serum IgG and SIgA in mucosal fluids in mice and nonhuman primates (16–18). These PspA-specific antibodies eliminated bacteria from lung lavage fluids, nasal washes, and the nasal passages (17). As a result, the cCHP-PspA nanogel vaccine protects against lethal or sublethal pneumococcal infections in immunized mice (17) and in mice that receive passively transferred serum from vaccinated macaques (16), as well as in pneumococcus-infected macaques (18). In addition, the vaccine antigen introduced by the cCHP nanogel did not migrate into the olfactory bulbs or brain in either murine or nonhuman primate models (16, 17); this is important evidence regarding the safety of a cCHP nanogel–based nasal vaccine delivery system.

P6 protein is a 16-kDa peptidoglycan-associated lipoprotein—one of the outer membrane proteins of NTHi—and is considered to be a potential vaccine antigen candidate for NTHi. In human studies, the amount of P6-specific SIgA correlates with the degree of inhibition of NTHi colonization of the nasopharynx and the incidence of recurrent otitis media (19, 20). Furthermore, nasal immunization of mice with P6 protein and cholera toxin, a classic and experimental mucosal adjuvant, induces P6-specific mucosal and systemic immune responses that clear NTHi from the nasal cavity after infection (21, 22). In addition, the P6 protein sequence is more than 90% conserved among NTHi strains at the nucleotide and amino acid levels, and the P6 protein is, therefore, a highly promising candidate antigen for the development of a universal NTHi vaccine (23).

Here, we investigated the quality and quantity of P6-specific immune responses, including protective efficacy, induced by nasally administered cCHP nanogel containing P6 protein (cCHP-P6). The cCHP-P6 nanogel nasal vaccine provided protective immunity against NTHi infection by inhibiting NTHi attachment to the nasal epithelial surface and preventing NTHi biofilm formation; systemic immunization did not lead to P6-specific IgA–mediated inhibition.

## Materials and Methods

### Mice

Female BALB/c mice (age, 7 to 8 weeks) were purchased from SLC (Shizuoka, Japan) or Kyudo Co. Ltd. (Saga, Japan). The mice were maintained in the experimental animal facility at the Institute of Medical Science of the University of Tokyo. All experiments were conducted in accordance with the guidelines provided by the Animal Care and Use Committees of the University of Tokyo and Oita University and were approved by the Animal Committee of the Institute of Medical Science of the University of Tokyo.

### P6 Antigen Construction and Recombinant Protein Purification

The P6 gene (GenBank accession no. AWP55884.1; amino acids 21–153) was synthesized by Takara Bio Inc. (Otsu, Japan). After digestion with the restriction enzymes *Nco*I and *Xho*I (Takara Bio Inc.), the gene was inserted into the pET-20b(+) vector (Novagen, Inc., Madison, WI, USA), which includes a C-terminal His tag. Rosetta2(DE3) pLysS-competent cells (Novagen, Inc.) were transformed with the P6-encoding plasmid in accordance with the manufacturer’s protocol. The resultant transformant was inoculated into lysogeny broth containing 100 μg/mL ampicillin and 34 μg/mL chloramphenicol and incubated with shaking at 37°C until the OD_600_ was 0.5 to 0.8. After induction with 0.4 mM isopropyl β-D-1-thiogalactopyranoside (Wako Pure Chemical Industries, Ltd., Osaka, Japan) and incubation at 37°C for 3.5 h, the cells were harvested by centrifugation at 5000*g* for 15 min at 4°C and then resuspended in 0.025 culture volume of Tris phosphate buffer containing 6 M urea. The desired protein was dialyzed against 6 M urea/500 mM NaCl/20 mM Tris containing 10 mM imidazole. The protein was then purified by means of Ni Sepharose 6 Fast Flow affinity chromatography (GE Healthcare Bio-Sciences K.K., Tokyo, Japan) followed by gel filtration on a Sephacryl S-100 HR column; GE Healthcare Bio-Sciences K.K.) in phosphate-buffered saline (PBS) containing 6 M urea. P6 fractions were collected and dialyzed step by step against 4 M urea–PBS, 2 M urea–PBS, 1 M urea–PBS, and PBS and kept at room temperature after passage through a 0.22-μm membrane. The protein concentrations of the purified P6 were determined according to the theoretical absorbance at a wavelength of 280 nm (absorbance 0.1% = 1.054), as determined from the amino acid sequence, and calculated by using the ProtParam tool (https://web.expasy.org/cgi-bin/protparam/).

### Preparation of cCHP Nanogel Vaccine and Immunization

A cationic type of nanogel (cCHP nanogel) was used for all experiments. The cCHP nanogel was synthesized as described previously (24). For the preparation of vaccine, the cCHP nanogel and recombinant P6 protein were mixed at a 1:1 molecular ratio and incubated for 1 h at 40°C. By using a *Limulus* test (Wako, Osaka, Japan), lipopolysaccharide contamination of the cCHP nanogel or recombinant P6 protein was confirmed to be less than 10 endotoxin units/mg protein. Mice were immunized intranasally with the cCHP-P6 without any adjuvant once weekly for 2 or 3 consecutive weeks (10 μg of P6 protein per immunization). Serum, nasal washes, and middle ear washes were obtained at 3, 5, 6, and 7 weeks after the first immunization. For collecting nasal wash samples, 100 μL of sterile PBS was flushed through the posterior choanae. Middle ear fluids were harvested by suspending 200 μL of sterile PBS in the middle ear (25). For systemic immunization, 20 μg of P6 protein precipitated with aluminum hydroxide was injected intramuscularly and then boosted with 10 μg of P6 in PBS at 2 and 5 weeks after the first immunization.

### Bacterial Strains and Infection

All of the clinical strains of NTHi were isolated from the nasopharynx of patients with effusive otitis media at Oita University Hospital (Oita, Japan); appropriate informed consent was obtained as described previously (PMID: 9665253). In brief, a Juhn Tym-Tap fluid collection aspirator (Xomed, Jacksonville, FL, USA) was inserted into the nasopharynx through the nose, and nasopharyngeal secretions were collected by aspiration. NTHi strains were isolated from nasopharyngeal secretions, stored at –80°C, and used in a manner that did not identify personal information (Grant-in-Aid for General Scientific Research (C), no. 06671724). Before use, all of the NTHi strains were grown overnight at 37 °C on chocolate agar plates prepared by using brain heart infusion broth. The number of bacteria was calculated by using the predetermined coefficient of 1 OD_600_ = 2 × 10^9^ colony-forming units (cfu)/ml; cells were pelleted and then diluted in PBS.

To evaluate vaccine efficacy, mice were challenged 1 week after the last immunization. The mice underwent intranasal pretreatment with 5 μl of 5% n-acetylcysteine, and then 5 μl of 0.5% Triton X-100 to increase susceptibility to infection. A sublethal dose (1 × 10^8^ cfu per mouse) of NTHi strain 76 diluted in 10 μL sterile PBS was then administered intranasally to each isofiurane-anesthetized mouse. Nasal washes were harvested 3 days after the sublethal challenge. For the preparation of nasal passage samples, nasal cavities were harvested 3 days after sublethal challenge, minced, and homogenized in PBS containing 1% saponin. Bacterial numbers in nasal washes or supernatant from nasal cavity homogenization were determined by counting colonies on chocolate agar plates.

### Antibody Titers

The endpoint titers of anti-P6 IgG or IgA from immunized mice were determined by using enzyme-linked immunosorbent assays (ELISAs), as described previously (17). Briefly, samples of serum, nasal wash, or middle ear wash were prepared as two-fold serial dilutions and loaded into a 96-micro-well plate (Nunc MaxiSorp Immuno; Thermo Fisher Scientific, Waltham, MA, USA) coated with 1 μg/mL recombinant P6 with bovine serum albumin. Horseradish peroxidase–conjugated goat anti-mouse IgG or IgA (dilution, 1:4,000) was used as a secondary antibody. Reactions were visualized by using the TMB Microwell Peroxidase Substrate System (XPL, Gaithersburg, MD, USA). The endpoint titer was expressed as the reciprocal log2 of the last dilution that gave an OD_450_ that was at least 0.1 unit greater than that of the negative control.

### IgA-Secreting Cells

Mononuclear cells were isolated from the nasal passages of immunized mice (5 × 10^5^ cells per mouse), and the numbers of P6-specific IgA producing cells were determined by using an enzyme-linked immunospot (ELISpot) assay. Briefly, 96-micro-well plates (MultiScreen; Merck, Darmstadt, Germany) were coated with 10 μg/mL recombinant P6 and incubated overnight at 4°C. Plates were washed three times with PBS and blocked for 1 h at 37 °C with RPMI 1640 medium supplemented with 10% fetal calf serum, 55 μM 2-mercaptoethanol, and 55 μg/ml penicillin–streptomycin. After the incubation, 2 × 10^5^ mononuclear cells isolated from the nasal passages of mice were added to each well and cultured at 37 °C, 5% CO_2_ for 6 h. Horseradish peroxidase–conjugated goat anti-mouse IgA (dilution, 1:1,000) was used as a secondary antibody. Spots were developed by adding 3-amino-9-ethylcarbazole (Merck) in 0.1 M sodium acetate buffer, pH 5.0, containing 0.05% H_2_O_2_ and incubating for 30 min at room temperature.

### Immunohistochemistry

Nasal cavity samples for confocal microscopy were prepared as described previously (26). Briefly, the nasal cavity samples were fixed in 4% (w/v) paraformaldehyde in PBS overnight at 4 °C with rocking, followed by soaking in 30% (w/v) sucrose in PBS overnight at 4 °C with rocking. The nasal cavity samples were then embedded in Super Cryoembedding Medium (Leica Microsystems K.K., Tokyo, Japan). For immunofluorescence staining, we prepared 10-μm-thick frozen sections by using a CryoJane Tape-Transfer System (Instrumedics, St. Louis, MO) and allowing the sections to air dry. Then the sections were treated with FITC-labeled anti-P6 antibody. After several washes, the specimens were mounted in VECTASHIELD mounting medium with DAPI (Vector Laboratories, Burlingame, CA, USA) and analyzed by using an LSM 800 confocal laser-scanning microscope (Zeiss, Oberkochen, Germany).

### Antibody Binding Assay

NTHi strain 76 was grown on a chocolate agar plate overnight at 37°C, and cells were collected in PBS and centrifuged at 10,000*g* for 5 min at 4°C, after which the cell pellet was diluted into FACS buffer. These NTHi cells (2 × 10^8^ cfu) were then incubated with nasal wash (equivalent to 50 μg protein input) for 1 h at room temperature, followed by staining with biotin-conjugated anti-mouse IgA (BioLegend, San Diego, CA, USA) and allophycocyanin–streptavidin (Tonbo Biosciences, San Diego, CA, USA). Flow cytometric analysis was performed by using an Attune NxT flow cytometer (Thermo Fisher Scientific).

### NTHi Adherence Assay

An *in vitro*, three-dimensional (3D) culture system reconstituted from healthy human primary airway tissue (MucilAir, Epithelix, Plan-les-Ouates, Switzerland) was used in the assay. FITC-labeled NTHi strain 76 cells (2.0 × 10^8^ cfu) were diluted in PBS and nasal wash (equivalent to 50 μg protein input) and incubated for 1 h at 37°C. The bacteria were then washed once with PBS, diluted in antibiotic-free medium, and added to the micro-titer plates of the cell culture system, which were placed a 5% CO_2_ incubator for 6 h at 37°C to allow the bacteria to adhere to the cells. Each well was then washed three times with PBS, and the cells were fixed in 4% (w/v) paraformaldehyde in PBS for 20 min at room temperature. This was followed by treatment with 0.1% Triton X-100 for permeabilization. The cells were stained with anti-β-tubulin (Merck), stained with rhodamine-conjugated anti-mouse IgG (Thermo Fisher Scientific) to visualize the cilia, mounted in VECTASHIELD mounting medium with DAPI (Vector Laboratories), and analyzed under an LSM 800 confocal laser-scanning microscope (Zeiss). By using COMSTAT2 software (27–29), we calculated the numbers of bacteria that were attached to epithelial surfaces (i.e., the biomass). This was done by determining the mean fluorescence of seven randomly selected fields of a slide image in which the FITC fluorescence signal exceeded the background fluorescence level of images obtained from 3D cultures without FITC-labeled NTHi. In addition, to deplete IgG, nasal washes were filtered through protein G Sepharose 4 Fast Flow (GE Healthcare Bio-Sciences K.K.). Residual amounts of IgG in the flowthrough samples were confirmed by using an ELISA, and the IgG-depleted samples were used at the same volumes as the crude nasal washes.

### NTHi Biofilm Formation Assay

The *in vitro* biofilm assay was performed as described previously (30). Briefly, NTHi bacteria were inoculated into the wells of an eight-well chamber slide (Thermo Fisher Scientific), each of which contained nasal washes from vaccinated mice, and incubated for 40 h at 37°C, 5% CO_2_. The medium in each well was changed every 12 h, and fresh nasal wash was added at each medium change. Biofilms were stained with LIVE/DEAD BacLight viability stain (Thermo Fisher Scientific) and fixed with 4% paraformaldehyde. Biofilms were visualized under a confocal microscope, and biomass values were quantified by using COMSTAT2 software (27–29).

### Statistical Analysis

Statistical analysis for comparisons among groups was performed by using either a two-tailed Student’s *t*-test or one-way ANOVA with a Tukey test. *P-*values less than 0.05 were considered significant.

## Results

### Nasal Immunization With cCHP Nanogel Carrying P6 Protein Induces P6-specific Mucosal Immune Responses

Competent *Escherichia coli* cells were transformed with the P6 plasmid, and the expressed His-tagged P6 recombinant protein was purified as a single band with a molecular weight of 15.5 kDa on SDS-PAGE (**Supplementary Figure S1**). Purified recombinant P6 protein was then incubated with the cCHP nanogel at a molecular ratio of 1:1 to prepare cCHP-P6 nanogel vaccine for the immunogenicity study. SDS treatment disrupts the cCHP-P6 vaccine, and the amount of P6 antigen released from the cCHP-P6 nanogel was evaluated by SDS-PAGE. More than 96% of the P6 antigen encapsulated in the cCHP nanogel was released without degradation, thus confirming the stability of the P6 antigen in the nanogel formulation (**Supplementary Figure S2**).

**Figure 1:**
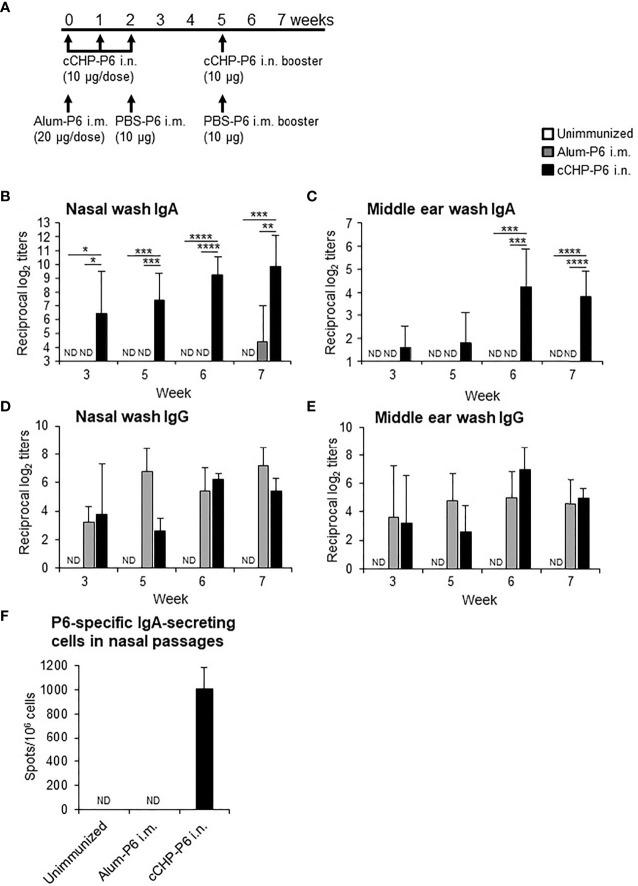
Intranasal immunization with cCHP-P6 nanogel nasal vaccine induces P6-specific antibody responses. **(A)** Wild-type female BALB/c mice were immunized with four doses of cCHP-P6 nanogel vaccine intranasally (i.n.) or with a single dose of alum-precipitated P6 protein by intramuscular (i.m.) injection, followed by two intramuscular doses of PBS-diluted P6 protein. The control group (unimmunized) received the same volumes of PBS both intranasally and intramuscularly. **(B–E)** Levels of P6-specific IgA in nasal washes **(B)** or middle ear washes **(C)** and P6-specific IgG in nasal washes **(D)** or middle ear washes **(E)** for each immunized group (cCHP-P6, Alum-P6, or unimmunized control) were determined by ELISA. **(F)** Numbers of P6-specific IgA-secreting cells in nasal passages were analyzed by using ELISpot. Data are representative of three independent experiments, and each group consisted of seven mice. ND, not detected in undiluted samples; n.s., not significant; *P < 0.05; **P < 0.01; ***P < 0.005; ****P < 0.001, one-way ANOVA with *post-hoc* Tukey test. Values are means ± 1 SD.

We then nasally immunized one group of BALB/c mice with the cCHP-P6 nanogel vaccine once weekly for 3 consecutive weeks (**Figure 1A**). Another group of BALB/c mice received a single intramuscular injection of P6 protein precipitated with aluminum hydroxide as an adjuvant (Alum-P6), followed by an intramuscular injection of P6 protein in PBS 2 weeks later. Mice in both groups received a booster immunization 3 weeks after the final immunization, and all animals received the same amount (40 μg) of the P6 protein. P6-specific IgA in nasal washes and middle ear washes (**Figures 1B, C****)** was induced only in the mice nasally immunized with the cCHP-P6 nanogel vaccine, and the levels were strongly enhanced after booster immunization. In contrast, Alum-P6–immunized mice had undetectable levels of P6-specific IgA in both nasal and middle ear secretions (**Figures 1B, C****)**. However, during the primary response, higher levels of P6-specific IgG were induced in the sera of the Alum-P6 group than in the cCHP-P6 group, although comparable levels of P6-specific IgG were induced in both groups after the booster dose (**Supplementary Figure S3**). Although P6-specific IgG was detected in the nasal and middle ear washes from both cCHP-P6 nanogel-vaccine- and Alum-P6-immunized mice (**Figures 1D, E****)**, reflecting plasma leakage into the mucosal fluids, the titers were similar between the immunization groups, and the levels were lower than those of the P6-specific IgA, especially in the nasal fluids. Moreover, P6-specific IgA–secreting cells were significantly more abundant in the nasal passages of the mice vaccinated with cCHP-P6 nasal vaccine but not in the nasal passages of the unimmunized controls or the mice intramuscularly immunized with Alum-P6 (**Figure 1F**).

### cCHP-P6 Nanogel Nasal Vaccine Induces Antibodies That Directly Bind to NTHi

**Figure 2:**
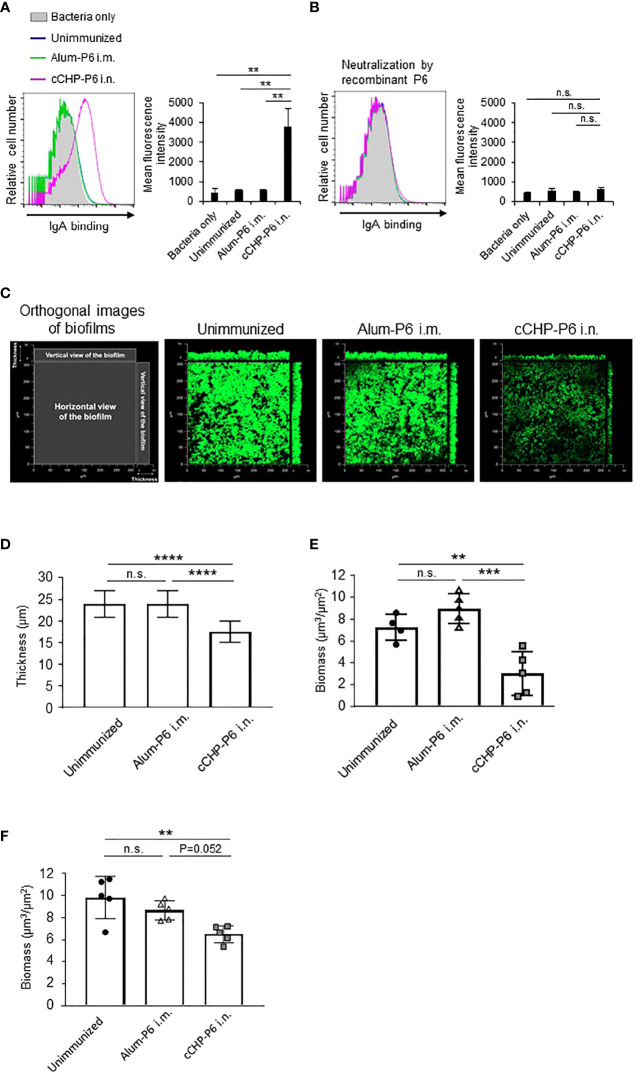
P6-specific IgA directly binds to NTHi and inhibits biofilm formation. **(A, B)** The binding activity of P6-specific IgA in nasal washes on the surface of NTHi strain 76 **(A)** or its activity after P6 protein neutralization **(B)** was determined by FACS analysis. The mean fluorescence intensity indicates antibody binding. **(C)** NTHi biofilms grown at 37°C, 5% CO_2_ for 40 h in a glass chamber slide with nasal washes of unimmunized or immunized mice. Biofilms were visualized with LIVE/DEAD BacLight viability stain and imaged with a confocal laser scanning microscope. **(D–F)** Biofilm thickness **(D)** and biomass values **(E)** treated with nasal washes or **(F)** biomass values treated with IgG-depleted nasal washes were quantified by using COMSTAT2 software. Data are representative of three independent experiments. **P < 0.01; ***P < 0.005; ****P  < 0.001; n.s., not significant; one-way ANOVA with *post-hoc* Tukey test. Values are means ± 1 SD.

Next, we used FACS to assess whether the P6-specific antibodies induced by the cCHP-P6 nanogel vaccine directly bound to NTHi. To this end, we incubated NTHi strain 76 cells with nasal washes from mice nasally immunized with cCHP-P6 or systemically immunized with Alum-P6. The nasal washes from the cCHP-P6–immunized mice contained P6-specific IgA antibodies that bound to NTHi strain 76 (**Figure 2A**). However, the nasal washes from Alum-P6–immunized mice did not contain any IgA antibodies that bound to NTHi strain 76—similar to the fluids from the unimmunized mice (**Figure 2A**). In addition, the vaccine-induced IgA binding to NTHi cells was mediated by the P6 on the surfaces of the bacteria, because the binding was abolished when the nasal washes were preincubated with recombinant P6 protein (**Figure 2B**).

### cCHP-P6 Nanogel Nasal Vaccine Suppresses NTHi Biofilm Formation

NTHi forms biofilms, which act as reservoirs of NTHi and cause infections in the upper and lower respiratory tracts (31, 32). NTHi biofilms increase resistance to antibiotics and trigger chronic and recurrent infections, including otitis media (31, 32). Because our results showed that P6-specific IgA bound effectively to the surfaces of NTHi cells (**Figure 2A**), we hypothesized that the antibody binding might physically inhibit NTHi biofilm formation. To analyze the effect of the P6-specific IgA on biofilm formation *in vitro*, NTHi strain 76 were grown in glass chamber slides for 40 h in the presence of nasal washes from mice nasally immunized with cCHP-P6. As controls, nasal washes from unimmunized or Alum-P6-immunized mice were tested also. Treatment of NTHi strain 76 cells with nasal washes from mice immunized with the cCHP-P6 nasal vaccine inhibited biofilm formation (**Figure 2C**), whereas nasal washes from Alum-P6 mice lacked inhibitory activity (**Figure 2C**). Incubation of NTHi strain 76 cells with nasal washes from the cCHP-P6 nasal vaccine group resulted in the generation of thin biofilms, and the biofilm biomass was significantly lower than that after incubation with nasal washes from Alum-P6–immunized or unimmunized control mice (**Figures 2D, E****)**. Because the nasal washes from the immunized group contained P6-specific IgG in addition to P6-specific IgA (**Figure 1D**), we also analyzed nasal washes from which total IgG was eliminated, to clarify the direct contribution of IgA to NTHi biofilm formation. Unlike the IgG-depleted nasal washes from Alum-P6-immunized mice, IgG-depleted nasal washes from the cCHP-P6 immunized mice decreased the biofilm biomass (**Figure 2F**). However, the inhibitory effect was slightly weaker with the IgG-depleted nasal washes from the cCHP-P6-immunized mice than with those containing IgG (**Figure 2E**), suggesting that the P6-specific IgG induced by the cCHP-P6 nasal vaccine may help to inhibit biofilm formation. Taken together, these results demonstrate that the P6-specific IgA induced by nasal immunization with cCHP-P6 reduced NTHi biofilm formation.

### cCHP-P6 Nanogel Nasal Vaccine Induces NTHi Clearance and Prevents NTHi Colonization of the Nasal Cavity after Nasal Infection

**Figure 3:**
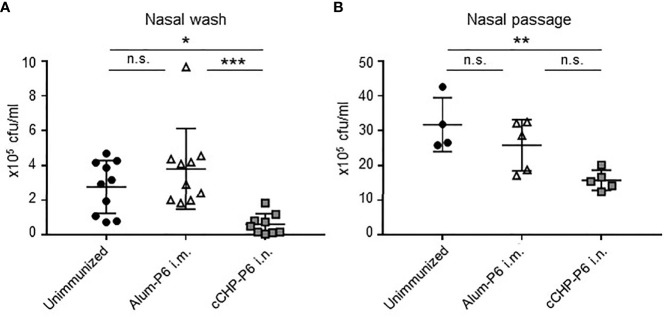
cCHP-P6 nanogel nasal vaccine provides protective immunity against NTHi infection. **(A, B)** Bacterial clearance from the nasal cavity was determined by counting the numbers of live NTHi in nasal washes **(A)** or nasal passages **(B)**. The concentration of NTHi was expressed as colony-forming units (cfu) per milliliter of sample. Data are representative of three independent experiments, with n = 10 for Unimmunized or Alum-P6 and n = 9 for cCHP-P6 **(A)** and n = 4 for Unimmunized and n = 5 in Alum-P6 or cCHP-P6 **(B)**. *P < 0.05; **P < 0.01; ***P < 0.005; n.s., not significant.

To investigate the protective efficacy of the cCHP-P6 nanogel vaccine, we performed intranasal challenge experiments. Mice that had been immunized nasally with cCHP-P6 or systemically with Alum-P6, as well as unimmunized control mice, were intranasally infected with NTHi strain 76. Three days after infection, the numbers of bacteria in nasal wash fluids and nasal passages from the immunized and unimmunized control mice were determined by counting colonies on chocolate agar plates. Bacterial numbers in the nasal washes were significantly lower in the cCHP-P6–immunized mice than in the Alum-P6–immunized or unimmunized control mice (**Figure 3A**). In the nasal passages, bacterial numbers were significantly lower in the cCHP-P6-immunized mice than in the unimmunized control mice (**Figure 3B**), but the difference between cCHP-P6-immunized- and Alum-P6-immunized mice was not significant. In particular, cCHP-P6 vaccination resulted in the marked clearance of NTHi from the nasal cavity (**Figure 3A**).

Although we noted elevated levels of P6-specific IgG, including Th2-related IgG1 and IgG2b subclasses (**Supplementary Figure S3**), in the sera of Alum-P6 vaccinated mice and passive leakage of P6-specific IgG into their nasal washes (**Figures 1D, E****)**, the IgG was ineffective at preventing NTHi colonization of the nasal tissue and failed to clear bacteria from the nasal cavity (**Figures 3A, B****)**. Indeed, P6-specific IgA–secreting cells were increased in number only in the nasal passages of cCHP-P6-immunized mice at 3 days after infection (**Supplementary Figure S4**), indicating that the clearance of NTHi was mediated by the P6-specific IgA induced in the nasal mucosa. Furthermore, tissue sections taken from the nasal cavities of unimmunized mice 3 days after NTHi intranasal challenge revealed that many NTHi organisms had colonized the mucosal epithelium and subepithelial regions (**Supplementary Figure S5**). NTHi colonization was markedly lower in the mice nasally vaccinated with cCHP-P6 than in the systemically immunized and unimmunized groups. These results demonstrate that the P6-specific IgA induced in nasal wash fluids by the cCHP-P6 nanogel nasal vaccine prevents NTHi infection by reducing the number of NTHi cells in the nasal tissue.

### cCHP-P6 Nanogel Nasal Vaccine–Induced IgA Binds to Different NTHi Clinical Isolates From Patients With Otitis Media

**Figure 4:**
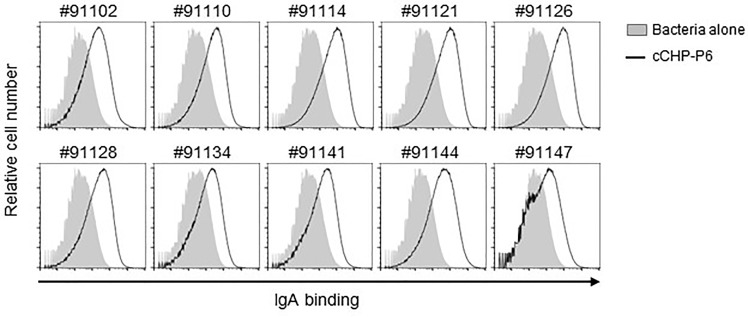
P6-specific IgA binds to diverse NTHi clinical isolates from patients with otitis media. Nasal washes collected from mice 2 weeks after the booster immunization were incubated with 10 NTHi clinical isolates of different strains (indicated by the numbers above the graphs). Antibody binding was detected by flow cytometric analysis using a secondary antibody. Data are representative of three independent experiments.

The universality of a vaccine is important for its clinical application. We incorporated the P6 protein as the vaccine antigen in our cationic nanogel (cCHP)–based nasal vaccine for otitis media because of its high immunogenicity, high expression levels, and high conservation at the nucleotide and amino acid sequence levels among different clinical isolates of NTHi (23). To analyze the breadth of the binding activity of P6-specific IgA induced by the cCHP-P6 nanogel nasal vaccine, we used FACS to analyze NTHi clinical isolates from the nasal washes of patients with otitis media. To this end, we incubated 10 NTHi clinical isolates with nasal washes from mice nasally immunized with cCHP-P6. As observed for NTHi strain 76 (**Figure 2A**)—itself a clinical isolate from the nasopharynx of a patient with otitis media—cCHP-P6 vaccine–induced IgA clearly bound to the surfaces of all 10 NTHi clinical isolates (**Figure 4**). Therefore, the cCHP-P6 nanogel nasal vaccine induces P6-specific mucosal IgA that binds to a broad variety of NTHi isolates from patients with otitis media.

### cCHP-P6 Nanogel Nasal Vaccine Inhibits NTHi Attachment to the Human Nasal Epithelial Surface

**Figure 5:**
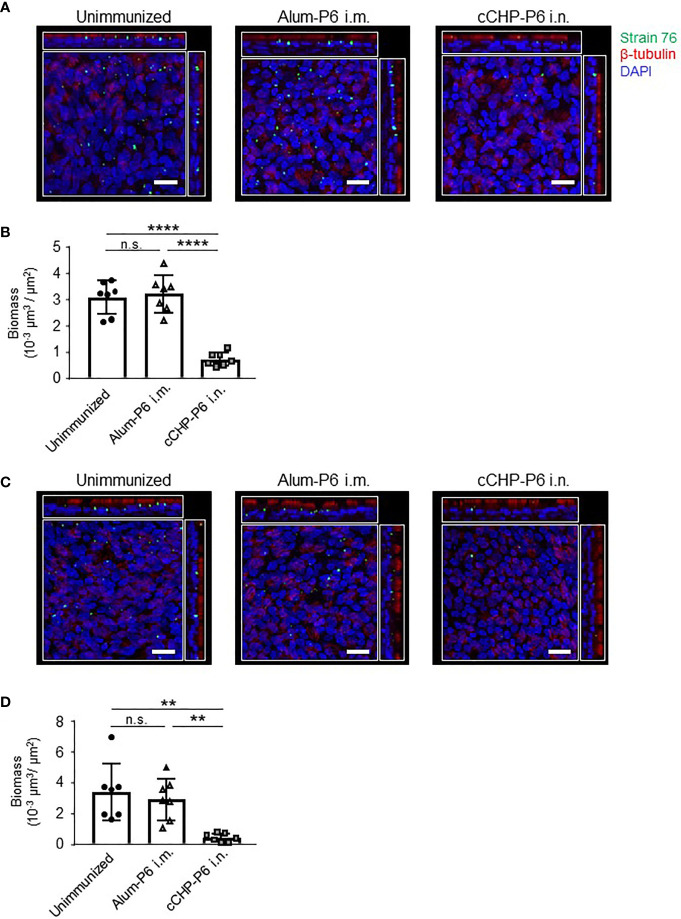
P6-specific IgA prevents the attachment of NTHi to human nasal epithelial cells. **(A–D)** A bacterial adherence assay was performed by using FITC-labeled NTHi strain 76. NTHi were pretreated with nasal washes **(A)** or IgG-depleted nasal washes **(C)** from immunized mice and then incubated with 3D human airway epithelium cultures at 37°C, 5% CO_2_ for 6 h. Epithelial cells were stained with β-tubulin (red) to visualize the cilia and with DAPI (4′,6-diamidino-2-phenylindole; blue) to visualize the nuclei. Images were obtained by confocal laser scanning microscopy. The number of NTHi organisms on the epithelial cells was determined according to the FITC signals in randomly selected fields of an image of the slide, and biomass values were quantified by using COMSTAT2 software **(B, D)**. Data are representative of three independent experiments. **P < 0.01; ****P < 0.001; n.s., not significant. Values are means ± 1 SD.

To invade the host, NTHi organisms first attach to nasal epithelial cells *via* several receptors, including platelet-activating factor receptor (8). Although the P6 protein itself does not mediate NTHi invasion, P6-specific IgA in the mucosal fluid directly binds to NTHi cells (**Figures 2A**, **4**), suggesting that the secretory antibodies enfold NTHi organisms and inhibit their attachment to mucosal epithelial cells. To test this notion and to determine whether this vaccine could be clinically useful, we investigated whether the cCHP-P6 nanogel nasal vaccine prevented NTHi attachment to human nasal epithelial cells. FITC-labeled NTHi strain 76 cells either left untreated or pretreated with nasal washes from immunized mice were co-cultured with 3D cultures of human airway epithelium, and the numbers of bacteria attached to the epithelial cells after 6 h of incubation were examined under a confocal microscope. Pretreatment with nasal washes from the cCHP-P6–nasally immunized mice significantly decreased the numbers of cells attached to epithelial cell surfaces, compared with pretreatment with nasal washes from unimmunized controls or mice intramuscularly immunized with Alum-P6 (**Figures 5A, B****)**. Likewise, IgG-depleted nasal washes from the cCHP-P6-immunized mice inhibited NTHi attachment to the human nasal epithelial cells (**Figures 5C, D****)**, indicating that P6-specific IgA, but not IgG, antibodies in nasal washes were responsible for this inhibition. These results suggest that the P6-specific IgA induced by nasal immunization with the cCHP-P6 nanogel vaccine prevents NTHi colonization of the mucosal surface by inhibiting NTHi attachment to human nasal epithelial cells.

## Discussion

For respiratory infections due to organisms such as NTHi, the nasal route is considered to be the most effective and logical vaccination strategy (10, 11). For example, nasal vaccination can induce both systemic and mucosal antigen-specific immune responses (12, 13). In contrast, injected vaccines effectively induce systemic antigen-specific immune responses but not mucosal immunity (33, 34). The antigen-specific SIgA produced at mucosal surfaces plays an important role in preventing invasion and colonization by pathogens at the site of infection (35). Because of these advantages of nasal vaccines, our groups have developed a cCHP-nanogel nasal vaccine delivery system and demonstrated the efficacy and safety of this system in both mice and non-human primates (16–18, 36, 37). In the present study, we developed a cCHP vehicle containing an NTHi nasal vaccine candidate antigen because a clinically desirable prophylactic vaccine for NTHi is currently unavailable. Recombinant P6 protein from NTHi is a particularly promising antigen candidate for an NTHi vaccine, because P6 has demonstrated high immunogenicity and is highly conserved among NTHi strains (23, 38, 39). We therefore incorporated P6 into cCHP and administered it intranasally to young adult mice. This resulted in the induction of high titers of P6-specific IgA and IgG in mucosal fluids and of IgG in serum (**Figures 1B–E** and **Supplementary Figure S3A**).

In our previous studies, we developed a cCHP-based pneumonia nasal vaccine, cCHP-PspA, which combined cCHP nanogel with a PspA recombinant protein that is a common surface antigen of *S. pneumoniae*. In mouse and non-human primate models, nasal immunization with cCHP-PspA effectively induced antigen-specific IgG in serum and SIgA in mucosal fluids (16–18). In addition, cCHP-PspA induced both Th2 and Th17 immune responses, which are associated with protective immunity (16–18). In our current study, cCHP nanogel combined with P6 protein similarly induced an effective Th2-type immune response supported by both IgG_1_ and IgG_2b_ antigen-specific antibodies (**Supplementary Figure S3B**).

In previous studies, the induction of P6-specific IgA in mucosal secretions and serum P6-specific IgG and IgA through nasal immunization with P6 protein required co-administration of mucosal adjuvants, such as CpG oligodeoxynucleotide, alpha-GalCer, and adamantylamide dipeptide (12, 13, 40). In those studies, nasal immunization with P6 protein alone elicited minimal to no antigen-specific immune responses and thus no effective protection against NTHi (12, 13, 40). In contrast, our cCHP-P6 nanogel nasal vaccine induced P6-specific mucosal IgA and serum IgG responses in the absence of a biologically active adjuvant, and the resulting P6-specific IgG titers were comparable to those induced by the intramuscularly administered vaccine containing alum, which is an extremely potent Th2 adjuvant (**Supplementary Figure S3A**). Moreover, booster immunization further increased P6-specific mucosal IgA and serum IgG levels (**Figures 1D, E** and **Supplementary Figure S3A**).

Here, we examined the efficacy of the cCHP-P6 vaccine by challenging vaccinated mice with an intranasal sublethal dose of NTHi strain 76. More NTHi was eliminated from the nasal cavities of cCHP-P6–nasally immunized mice than of Alum-P6–intramuscularly immunized mice or unimmunized controls (**Figures 3A, B**, and **Supplementary Figure S5**). In previous investigations, the P6-specific IgG induced in mice or chinchillas that received P6 by systemic immunization or in naturally infected humans had strong bactericidal activity and contributed to host defense against NTHi infection (41–43). We demonstrated here that—in addition to this bactericidal activity of the P6-mediated immune response in the systemic compartment—the P6-specific mucosal IgA induced by our cCHP-P6 nanogel nasal vaccine helped to reduce biofilm formation (**Figures 2C–F**). In contrast, nasal washes from Alum-P6 vaccinated mice only weakly suppressed biofilm formation (**Figures 2C–F**), perhaps because of the very low levels of P6-specific IgA in their nasal fluids despite the presence of P6-specific IgG owing to plasma leakage (**Figures 1D, E****)**. Thus, when we examined the concentrations of P6-specific antibodies in nasal washes from intramuscularly immunized mice, the IgG levels ranged from 3 to 7 in log2-scale of reciprocal titers, whereas IgA isotype was undetectable (**Figures 1B, D****)**. In contrast, nasal washes from nasal cCHP-P6 immunized mice had concentrations of 3 to 9 and 3 to 6 in log2-scale reciprocal titers for P6-specific IgA and IgG isotypes, respectively (**Figures 1B, D****)**.

In addition to an interaction between phosphorylcholine and platelet-activating factor receptor, which is known to facilitate bacterial adhesion to the epithelium and host invasion, several adhesion factors, including Pili, HMW1/HMW2, Hap, and Hia, mediate the interaction of NTHi with host-cell extracellular matrix proteins such as laminin, fibronectin, and collagen IV to promote the aggregation of NTHi bacteria, their adherence to epithelial cells, and their entry into these cells (8). NTHi biofilms contribute to bacterial persistence and pathogenesis in the respiratory tract and pharynx, resulting in increased antibiotic resistance and chronic and recurrent otitis media (9, 44). Our current study provides evidence that P6-specific mucosal IgA reduces NTHi biofilm formation (**Figures 2C–F**).

Unlike adhesive factors such as Pili, the P6 protein is not directly involved in the adhesion of NTHi to the mucosal epithelium. Therefore, we speculate that the reduction of NTHi biofilm formation by P6-specific antibodies is due to steric hindrance caused by direct antibody binding to the surface of NTHi. Another component of the inhibition mechanism might be associated with the functional nature of P6. So far, the function of P6 protein in NTHi bacteria has not been clarified, but Pal protein—an *E. coli* homolog of P6—reportedly binds both peptidoglycan and extracellular membrane proteins and contributes to cell-wall stability (45–47). Indeed, a P6-deletion strain of NTHi proliferates more slowly than the wild-type strain, with morphological changes including increased cell size and decreased cell-wall integrity compared with those in wild-type organisms (48). Therefore, the inhibition of P6-mediated cell-wall integrity and cell growth by specific antibodies during NTHi cell division might be linked to the suppression of biofilm formation. The details of this mechanism need to be elucidated in future investigations.

Clinical reports have indicated an inverse correlation between P6-specific antibody levels and NTHi colonization of the nasopharynx or development of recurrent otitis media in children (38, 49); these findings indicate that P6 is a reasonable target for NTHi vaccination. In addition to having excellent immunogenicity, P6 is abundantly expressed in all NTHi strains, with extremely high (>90%) conservation at the nucleotide and amino acid sequence levels (23). In our hands, the 11 clinical isolates of NTHi, including strain 76, revealed high conservation, although single-amino-acid substitutions were present at three positions (**Supplementary Figure S6**). In addition, our present study showed that cCHP-P6–induced mucosal IgA directly bound to the surfaces of all 11 of the NTHi clinical isolates—each from a different patient with otitis media—without exception (**Figure 4**). In addition, through its binding activity, P6-specific mucosal IgA is supposed to capture NTHi bacteria and thus might influence the interaction between NTHi organisms and host-cell surfaces. Indeed, the P6-specific mucosal IgA induced by the cCHP-P6 nanogel nasal vaccine effectively inhibited the attachment of NTHi bacteria to primary human nasal epithelial cells in culture (**Figures 5A, B**). This inhibition is mediated by P6-specific mucosal IgA, and not by P6-specific IgG, because the inhibition was still observed when nasal washes depleted of IgG were used (**Figures 5C, D****)**. Moreover, nasal washes obtained after systemic (e.g., intramuscular) immunization did not prevent NTHi attachment (**Figures 5A–D**).

In conclusion, the current study demonstrated that a cCHP-nanogel nasal vaccine targeting P6 protein provides NTHi-specific protective immunity by preventing NTHi colonization of the surface of the nasal mucosa through a reduction in biofilm formation and epithelial-cell attachment of NTHi. Therefore, this cCHP-P6 nasal vaccine is a potential universal vaccine for NTHi infectious diseases, including otitis media in young children, and its ability to meet this unmet clinical need should be investigated further.

## Data Availability Statement

The original contributions presented in the study are included in the article/**Supplementary Material**. Further inquiries can be directed to the corresponding authors.

## Ethics Statement

The animal study was reviewed and approved by Animal Committee of the Institute of Medical Science of the University of Tokyo.

## Author Contributions

RN-O, HM, and YY were responsible for study conceptualization. HM, SU, TH, YU, TM, TY, SS, KA, and YK were responsible for study methods and investigations. YU was responsible for statistical analysis. RN-O, YY, and HK were responsible for writing the manuscript. MS, KA, KF, and YK were responsible for reviewing and editing the manuscript. RN-O, YY, and HK were responsible for funding acquisition. All authors contributed to the article and approved the submitted version.

## Funding

This research was supported by the Translational Research Program “Strategic Promotion for Practical Application of Innovative Medical Technology (TR-SPRINT)” from the Japan Agency for Medical Research and Development (AMED). The sponsor had no control over the interpretation, writing, or publication of this work.

## Conflict of Interest

YY and HK are directors and founders of HanaVax Inc. RN-O, S-IS, KA, and YK are scientific advisors of HanaVax Inc.

The remaining authors declare that the research was conducted in the absence of any commercial or financial relationships that could be construed as a potential conflict of interest.

## Publisher’s Note

All claims expressed in this article are solely those of the authors and do not necessarily represent those of their affiliated organizations, or those of the publisher, the editors and the reviewers. Any product that may be evaluated in this article, or claim that may be made by its manufacturer, is not guaranteed or endorsed by the publisher.
