# Supplementary material for: Induction of Mucosal IgA–Mediated Protective Immunity Against Nontypeable Haemophilus influenzae Infection by a Cationic Nanogel–Based P6 Nasal Vaccine
Source: Front Immunol. 2022 Jul 6;13:819859. doi: 10.3389/fimmu.2022.819859 (PMC9299436; doi:10.3389/fimmu.2022.819859)
Supplement: Supplementary file 1 [file DataSheet_1.pdf]

A

B

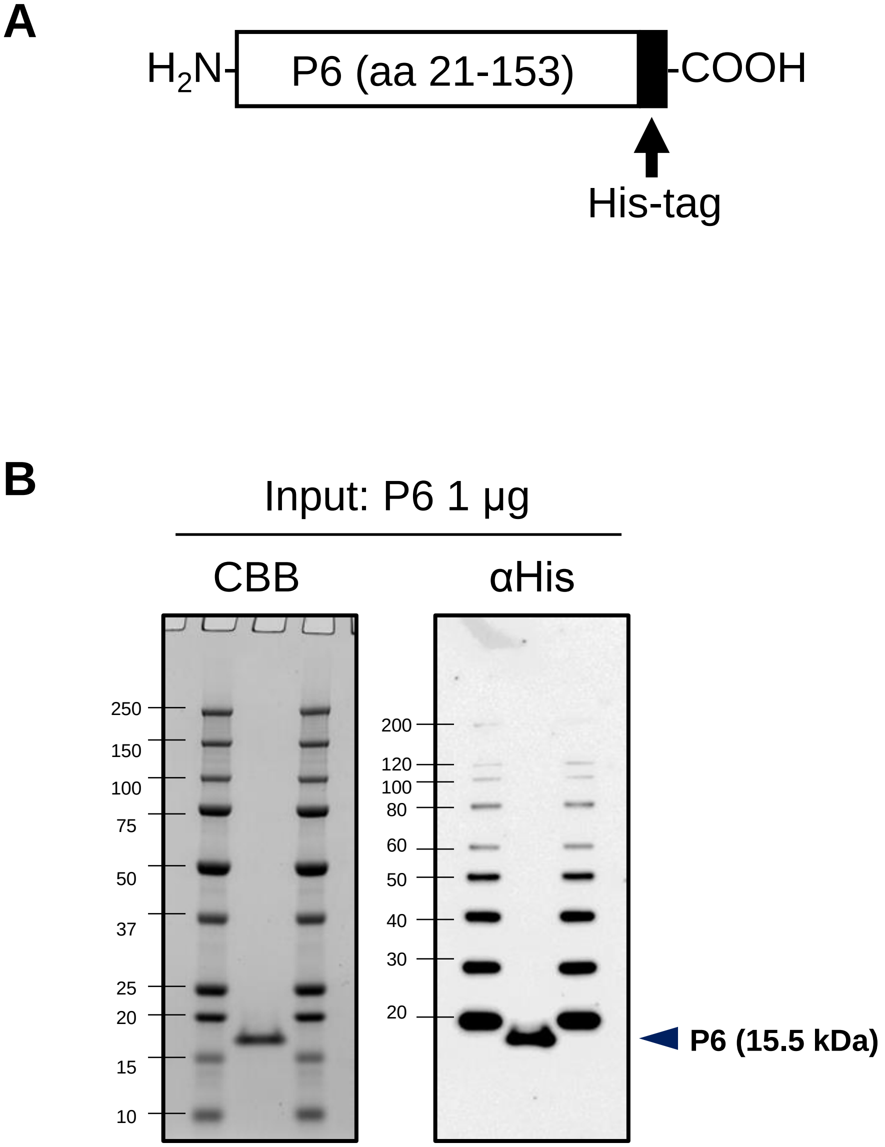

Supplementary Figure S1. P6 protein structure and purified recombinant P6 protein. (A) P6 vaccine antigen structure according to the nucleotide sequence from GenBank (accession no. AWP55884.1). A histidine tag was added at the C-terminus of the P6 protein. **(B)** Purified P6 protein was stained as a single band with a molecular weight of 15.5 kDa by using Coomassie Brilliant Blue (CBB) and was also identified by means of western blotting with anti-His-tag antibody.

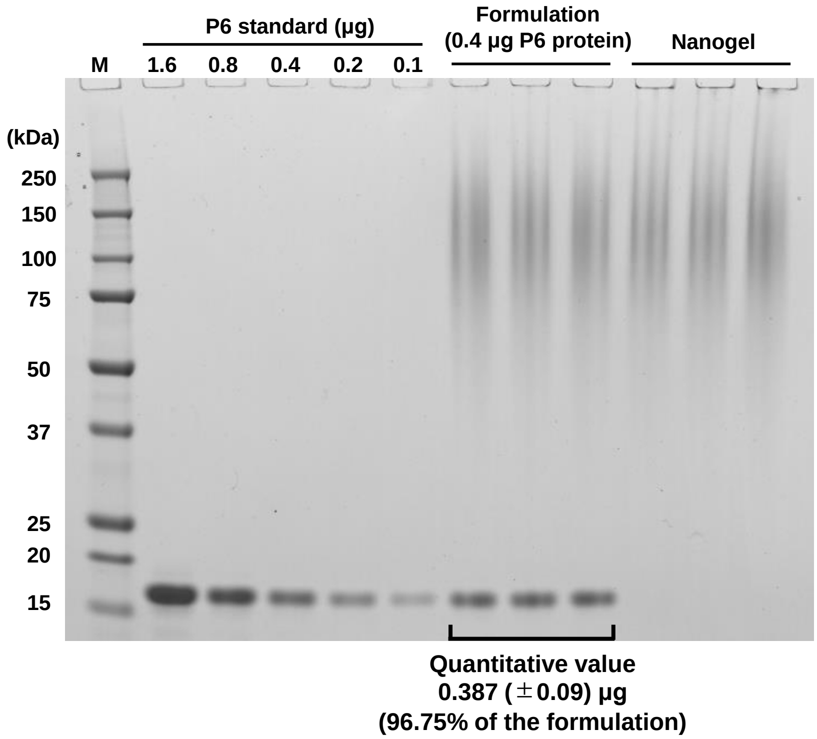

Supplementary Figure S2. Total P6 content in the cCHP−P6 vaccine formulation. The total amount of P6 protein released from the cCHP−P6 formulation was measured quantitatively through SDS−PAGE followed by protein staining and a densitometric analysis. The gel contained three replicate samples of the formulation and of the nanogel itself. M is a molecular marker. The quantitative value shown is the mean ± 1 SD of three wells.

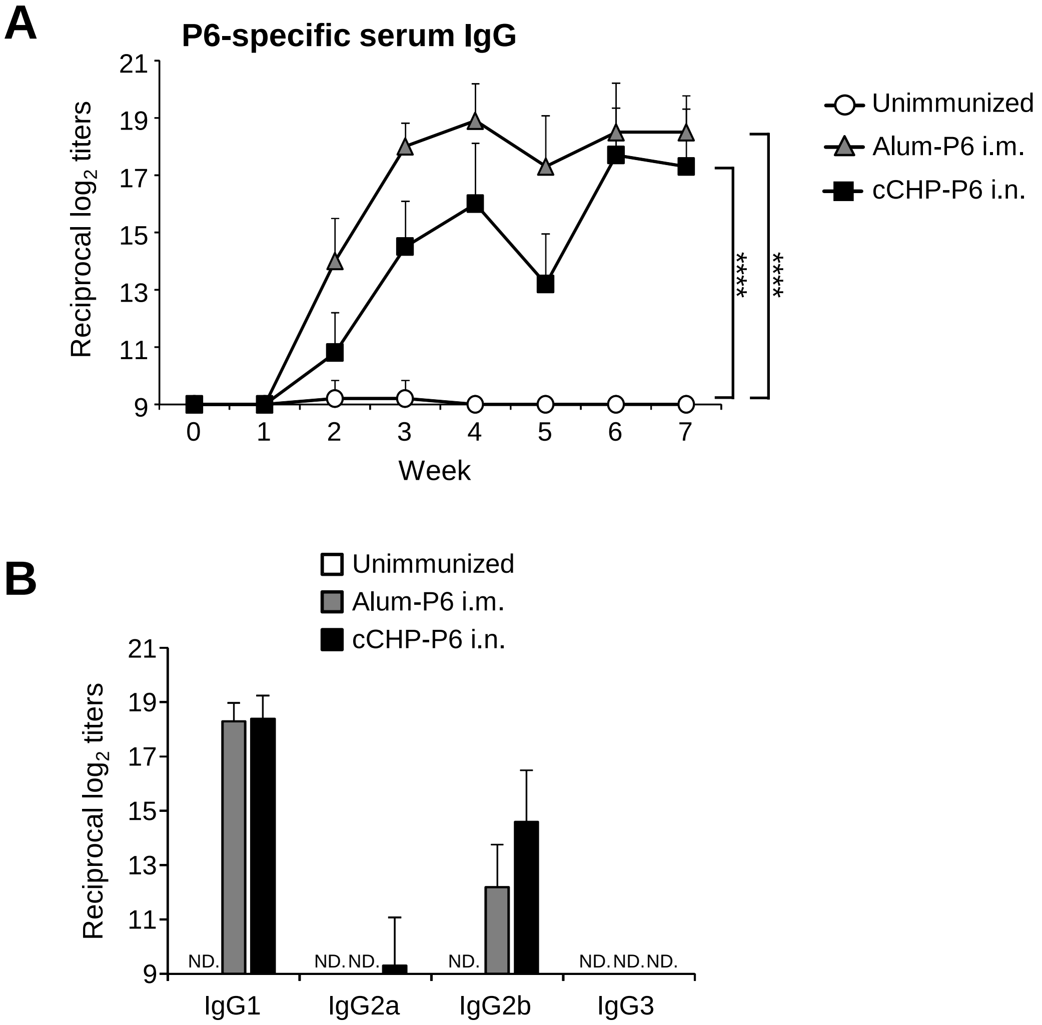

Supplementary Figure S3. P6-specific serum IgG is efficiently induced after cCHP-P6 intranasal immunization or P6-Alum intramuscular immunization. (A) Levels of P6-specific IgG or **(B)** the P6-specific IgG subclasses for IgG1, IgG2a, IgG2b, and IgG3 in sera collected from cCHP-P6 intranasally (i.n.) immunized, P6-Alum intramuscularly (i.m.) immunized, or unimmunized mice were determined by using ELISA. Data are representative of three independent experiments, and each group consisted of seven mice. **** $P < 0.001$. Values are means ± 1 SD.

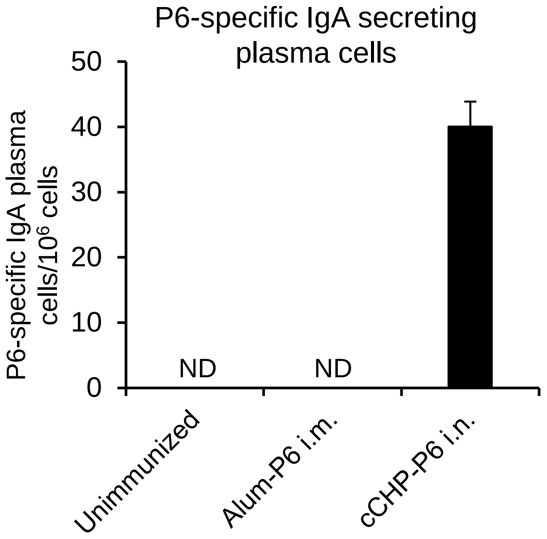

Supplementary Figure S4. P6-specific IgA-secreting cells are increased in nasal tissues of cCHP-P6 immunized mice after NTHi nasal infection. The numbers of P6-specific IgA secreting cells in nasal passages after NTHi infection were analyzed by using ELISpot. Data are representative of three independent experiments, and each group consisted of three mice. ND, not detected in undiluted samples.

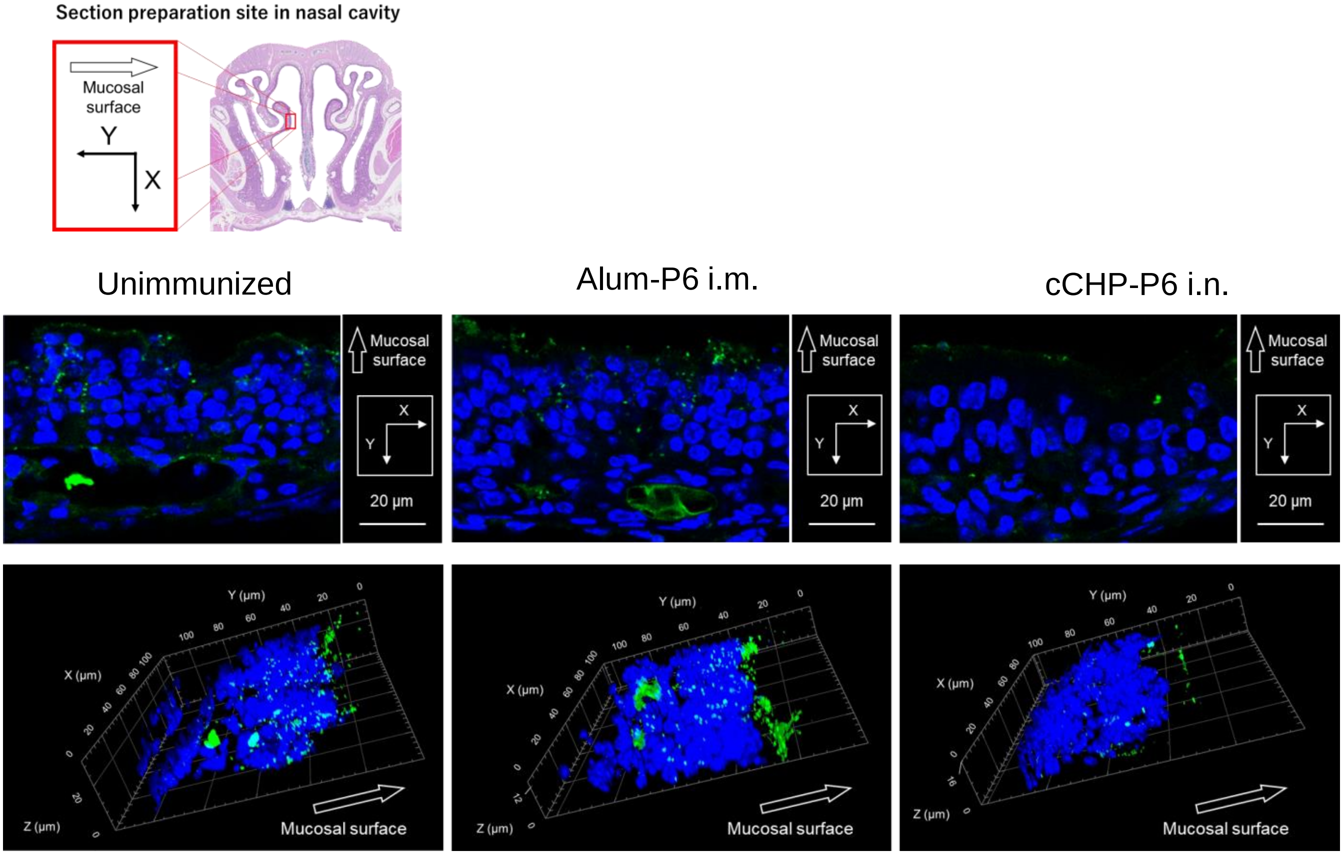

Supplementary Figure S5. NTHi localization in mouse nasal tissues. Localization of NTHi after nasal infection was detected by using immunofluorescence assays. Tissue sections were prepared and stained with anti-P6 (FITC [green]) and DAPI (blue). Green spots indicate NTHi. Data are representative of two independent experiments (n = 2 or 3 mice per group).

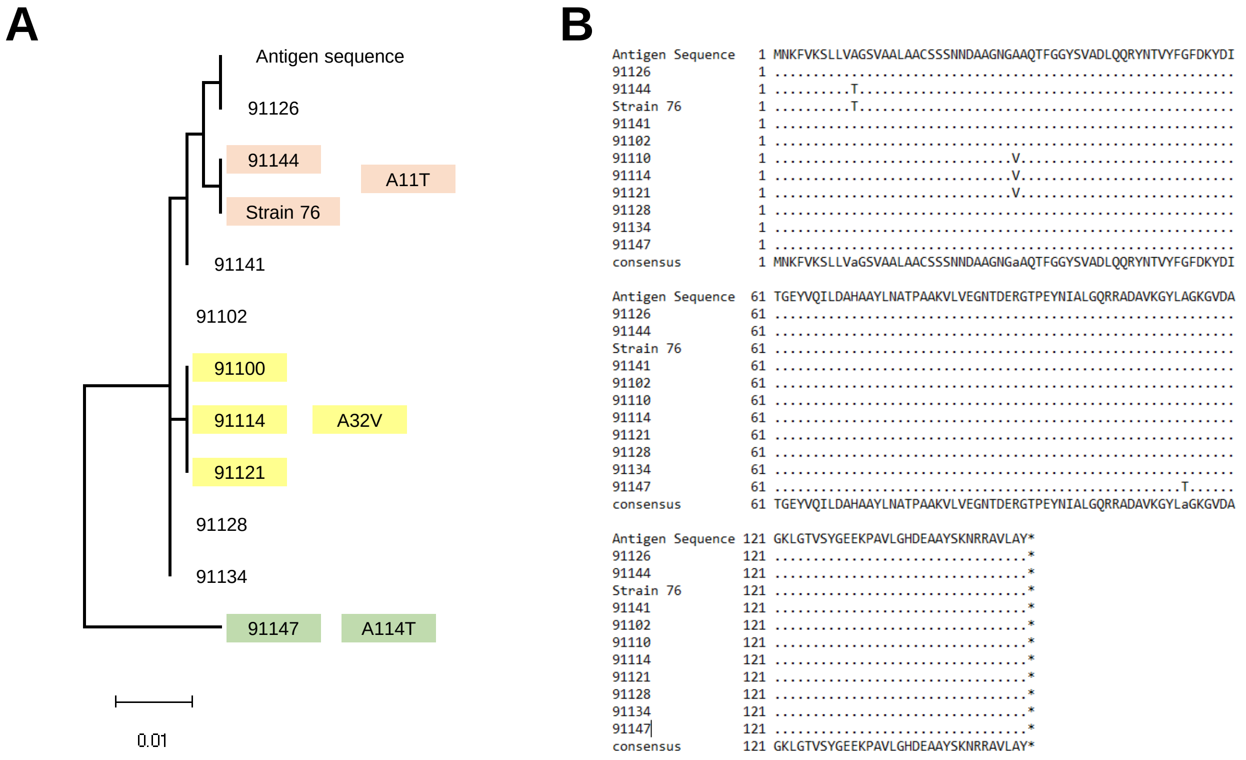

Supplementary Figure S6. The amino acid sequence of P6 protein is highly conserved in the NTHi clinical isolates, with the exception of three single-amino-acid substitutions. (A) A phylogenetic tree based on the nucleic acid sequences of the P6 genes from the indicated NTHi clinical isolates was constructed by using the maximum likelihood method and the Tamura–Nei model. **(B)** Amino acid sequences of the NTHi clinical isolates, the antigen sequence used in this research, and the consensus sequence are shown. Amino acids are marked at the point where they differ from the consensus sequence.
